# Impact of smoking reduction on lung cancer risk in patients with COPD who smoked fewer than 30 pack-years: a nationwide population-based cohort study

**DOI:** 10.1186/s12931-024-02741-1

**Published:** 2024-03-18

**Authors:** Sun Hye Shin, Taeyun Kim, Hyunsoo Kim, Juhee Cho, Danbee Kang, Hye Yun Park

**Affiliations:** 1 Division of Pulmonary and Critical Care Medicine, Department of Internal Medicine, Samsung Medical Center, Sungkyunkwan University School of Medicine, 81 Irwon-ro, Gangnam-gu, Seoul, 06351 South Korea; 2 Department of Clinical Research Design and Evaluation, SAIHST, Sungkyunkwan University, 81 Irwon-ro, Gangnam-gu, Seoul, 06351 South Korea; 3 Department of Epidemiology and Welch Center for Prevention, Epidemiology, and Clinical Research, Johns Hopkins University Bloomberg School of Public Health, Baltimore, MD USA

**Keywords:** Smoking, COPD, Lung cancer

## Abstract

**Background:**

The effects of smoking reduction on the incidence of lung cancer in patients with chronic obstructive pulmonary disease (COPD) are not well known. This study aimed to investigate the effects of changes in smoking habits after COPD diagnosis on lung cancer development in patients who smoked less than 30 pack-years.

**Methods:**

This nationwide retrospective cohort study included 16,832 patients with COPD who smoked less than 30 pack-years at the time of COPD diagnosis. Based on changes in smoking habits in the health screening examination data, smokers were categorized into three groups: quitters, reducers, and sustainers. The primary outcome was the risk of lung cancer development, which was estimated using the Cox proportional hazards model. We also modelled the amount of smoking reduction as a continuous variable.

**Results:**

During a median follow-up of 4 years, the cumulative incidence of lung cancer was the highest among sustainers, followed by reducers and quitters. Compared with sustainers, reducers (adjusted HR 0.74, 95% CI:0.56–0.98) and quitters (adjusted HR 0.78, 95% CI:0.64–0.96) had a significantly lower risk of lung cancer. Incidence of lung cancer showed a decreasing trend with a decreasing amount of smoking (P for linearity < 0.01).

**Conclusions:**

In patients with COPD who smoked less than 30 pack-years, smoking reduction and cessation lowered the risk of lung cancer.

## Background

Chronic obstructive pulmonary disease (COPD) is a chronic inflammatory lung disease characterized by persistent respiratory symptoms and airflow limitation [1]. Comorbidities are common in patients with COPD and can contribute to symptom severity and disease progression [2]. Lung cancer is one of the most frequent and burdensome comorbidities and the leading cause of death in patients with COPD [3]. Cigarette smoking is a well-known common risk factor for both COPD and lung cancer, and it further increases the risk of lung cancer in patients with COPD [4]. Indeed, a large national cohort study showed that smokers with COPD and never smokers with COPD had approximately 6.2 and 2.6 times the incidence of lung cancer, respectively, compared with never smokers without COPD [4]. Accordingly, the attainment of complete smoking cessation is the most effective way to reduce the risk of lung cancer [5, 6]. Regarding smoking reduction, although the prospective cohort study for cardiovascular disease (from mid-1970s to 2003) found no difference in lung cancer related mortality between sustainers and > 50% reducers [7], accumulated general population studies showed that there is a dose-dependent effect of the amount of smoking reduction [5, 6, 8] and the duration of smoking cessation [9] on the risk of lung cancer.

In patients with COPD, few studies have investigated the effect of changes in smoking habits on lung cancer-related outcomes. The Lung Health Study reported the impact of changes in smoking habits on lung cancer mortality in patients with COPD [10]. However, based on smoking history, the patients in that study were categorized as sustainers, quitters, and intermittent quitters, and the dose-dependent association between smoking habit change and lung cancer mortality could not be measured [10]. A single-center retrospective study in China found a reduction in all-cause mortality in patients with COPD who quit smoking compared with those who continued smoking; however, there was no significant difference in lung cancer mortality between the two groups, which might be explained by the small sample size (*n* = 204) and number of lung cancer mortality cases (*n* = 15) [11]. No study has investigated the dose-dependent effects of smoking reduction on the incidence of lung cancer in patients with COPD.

Landmark studies have shown that lung cancer mortality was reduced by lung cancer screening using chest computed tomography in a high-risk population with a minimum of 15 to 30 pack-years of smoking history [12, 13]. In contrast, those with less smoking exposure have not received enough attention despite growing evidence showing that smokers with less than 30 pack-years of exposure also have a considerable risk of developing lung cancer [14, 15]. Although the recent guideline from the United States on lung cancer screening have expanded the selection criteria [16], still there is a scarcity of data regarding the lung cancer development in individuals with less smoking exposure. Furthermore, given that the COPD itself is regarded as a risk factor in selecting the candidate for lung cancer screening [17, 18], it would be of interest to estimate the degree of lung cancer risk reduction in patients with COPD by the amount of smoking reduction. In this context, this nationwide cohort study aimed to evaluate the impact of smoking reduction on the incidence of lung cancer after a COPD diagnosis in smokers using less than 30 pack-years, by categorizing them as quitters, reducers, or sustainers.

## Methods

### Data source

This retrospective cohort study used data from the Korean National Health Insurance System (K-NHIS) database. The K-NHIS database represents the entire South Korean population. The K-NHIS claims database contains information on patient demographics, medical treatments, procedures, prescription drugs, diagnostic codes, and hospital use. Diagnoses in the K-NHIS database were based on the International Classification of Diseases, 10th revision (ICD-10). The K-NHIS regularly audits ICD-10 codes, procedure records, and prescription records to avoid unnecessary medical expenses. Additionally, the K-NHIS claims database includes data from the National Health Screening Examination, a standardized health screening program provided to all insured persons every 2 years [19]. The participation rate of the target population in the National Health Screening Examination is approximately 76% [19]. The Health Screening Examination data includes a self-administered questionnaire on medical history, lifestyle habits, anthropometric measurements, and laboratory tests [19]. Health examination facilities are designated and overseen for quality control according to relevant national laws and regulations. Further details regarding the NHIS database and health examinations are described elsewhere [19, 20].

### Study population

Our database included all patients with COPD aged ≥ 40 years between January 1, 2014 and December 31, 2019. COPD was defined as the presence of the J43-J44 code (except J43.0) (ICD-10) and the prescription of COPD medication at least twice within 1 year. Medications for COPD include long-acting muscarinic antagonists (LAMAs), long-acting beta-2 agonists (LABAs), inhaled corticosteroids (ICSs) plus LABAs, short-acting muscarinic antagonists (SAMAs), short-acting beta-2 agonists (SABAs), methylxanthines, systemic beta agonists, and phosphodiesterase-4 (PDE-4) inhibitor [4, 21, 22].

**Fig. 1:**
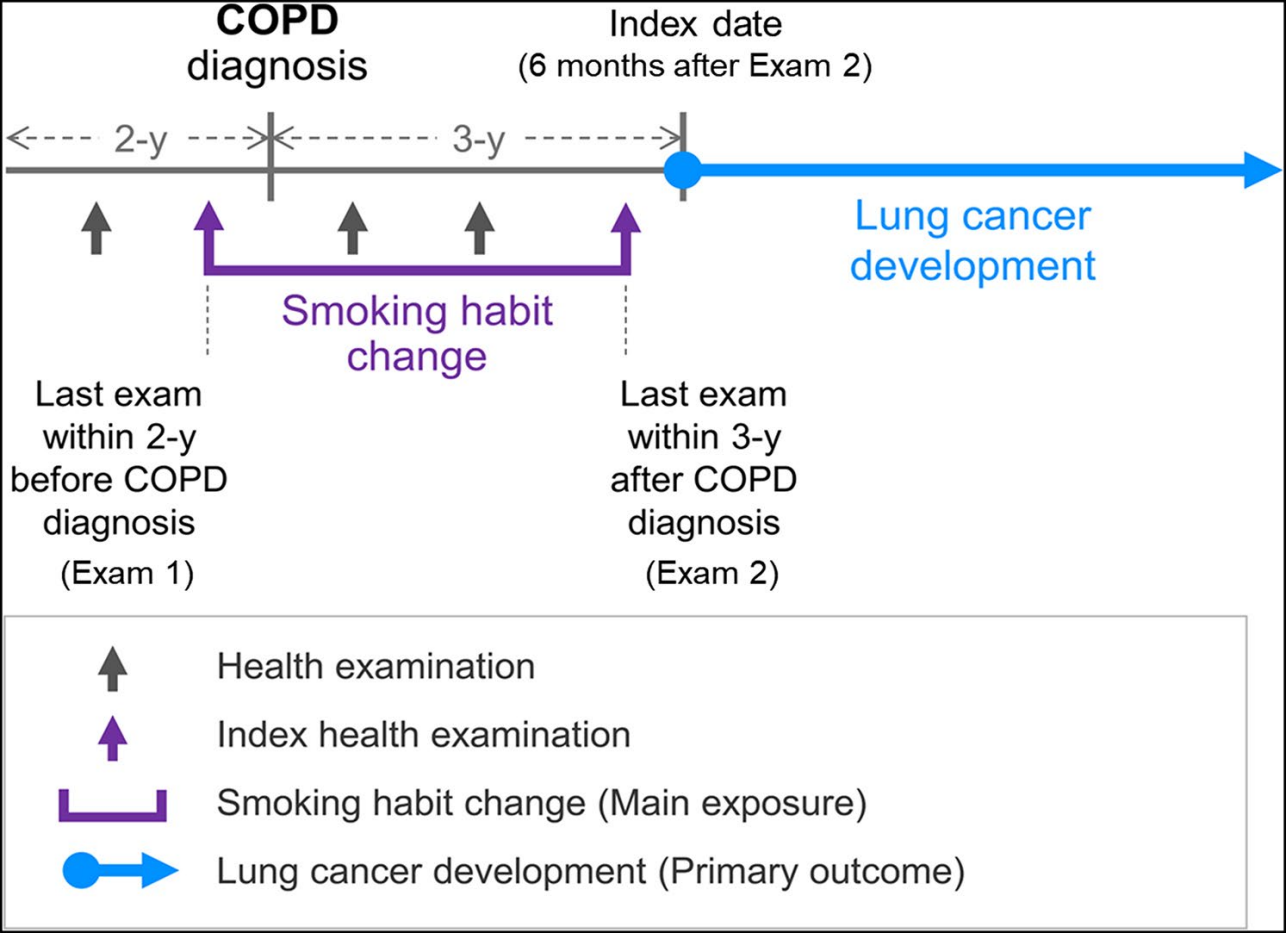
Study flow process

As the purpose of this study was to evaluate the effects of smoking reduction and cessation after COPD diagnosis on lung cancer development, we included only patients who were smoking before the diagnosis of COPD (Exam 1, *N* = 45,271). Among them, 38,077 patients had health examination data within 3 years of the date of COPD diagnosis (Exam 2). We excluded 2,816 participants who had cancer before the Exam 2. Furthermore, to minimize potential reverse causality, we further excluded 1,232 participants who developed any cancer or died within the first 6 months of follow-up from the Exam 2 index date. To focus on low-dose smokers, 16, 832 participants who had smoked less than 30 pack-years were selected. A period of 3 years was chosen *a priori* based on previous literature as well as the anticipated sample size and follow-up duration [23, 24]. A brief summary of the study process is shown in Fig. 1.

The Institutional Review Board of Samsung Medical Center approved the study (approval no.2022-09-022) and waived the requirement for informed consent because the K-NHIS data were de-identified.

### Assessment of smoking habit

Smoking status was assessed using a self-reported questionnaire during each of the last examinations within 2 years before COPD diagnosis (Exam 1) and within 3 years after COPD diagnosis (Exam 2). Current smokers were questioned about their duration of smoking and the mean number of cigarettes smoked per day. According to the smoking intensity in Exam 1, smokers were defined as light (< 10 cigarettes per day), moderate (10–19 cigarettes per day), and heavy (≥ 20 cigarettes per day) smokers [5].

In this study, changes in cigarette smoking intensity were identified based on relative changes in the number of cigarettes smoked per day. Participants were categorized into three groups based on the relative change in smoking intensity between Exam 1 and Exam 2: quitters, reducers, and sustainers, based on the definitions used in previous studies [25]. Quitters were defined as those who had completely stopped smoking (i.e., current smokers in Exam 1 who became former smokers in Exam 2). The reducer group included those who decreased their number of cigarettes consumed per day by 20% or more. Additionally, the reducer group was divided into subcategories to evaluate the possible dose responsiveness of smoking reduction: >50% reducers were defined as those who decreased the number of cigarettes per day by 50% or more, while 20–50% reducers were defined as those who decreased the number of cigarettes per day by 20–50%. Sustainers were defined as those who maintained (increased or decreased by less than 20%) the number of cigarettes they consumed per day.

### Covariables

Residential areas and income levels were obtained from insurance eligibility. The residential areas were categorized as metropolitan cities (Seoul, Busan, Daegu, Daejeon, Gwangju, Incheon, and Ulsan) and others. Income levels were categorized as Medical Aid, ≤ 30th, 30–70th, or > 70th percentile. Data on alcohol consumption, physical activity, and body mass index (BMI) were collected during Exam 2.

Severe COPD exacerbation was defined as hospitalization or emergency room visit with one of the following ICD-10 codes as the principal or secondary diagnosis: COPD (J43.X [except J43.0] or J44.X), COPD-related disease (pneumonia [J12.X–J17.X], pulmonary thromboembolism [I26, I26.0, or I26.9], dyspnea [R06.0], or acute respiratory distress syndrome [J80]), and a prescription for systemic steroids or antibiotics at the same visit [26]. Comorbidities during the year prior to Exam 2 were obtained from claims data defined using ICD-10 codes and summarized using the Charlson comorbidity index (CCI) [27]. In addition to CCI, we included pulmonary tuberculosis (ICD-10: A15, A16, and B90.9), interstitial lung disease (ICD-10: J84), bronchiectasis (ICD-10: J47), and pneumonia (ICD-10: J11 ~ J18, J69) using insurance claims data during a 1-year look-back period from Exam 2.

### Outcomes

The primary endpoint was lung cancer incidence. Lung cancer was defined as the presence of a cancer-specific insurance claims code (V193) with a C33 or C34 code, which is the ICD-10 code for lung cancer. In Korea, once a person receives a cancer diagnosis, he/she is registered with the National Cancer Registry with a specific code that indicates to the system that the person has been diagnosed with cancer and is receiving special insurance benefits.

### Statistical analysis

The incidence rates were calculated as the number of events per 100 person-years of follow-up. We used the Kaplan-Meier curve to evaluate the cumulative incidence of lung cancer by group. In the figure, age was used as a timescale. Hazard ratios (HRs) and the corresponding 95% confidence intervals (CIs) of the outcomes for each group were calculated using Cox proportional hazards models. The proportionality of the hazards was confirmed by visual inspection of the log-minus-log plots and Schoenfeld residuals.

The models were adjusted for age, sex, BMI, residential area, income, regular physical activity, smoking pack-years (Exam 1), ICS prescription within 1 year of Exam 2, history of severe exacerbation within 1 year of Exam 2, and comorbidities within 1 year of Exam 2. Covariables were selected *a priori* based on their possible association with smoking habits and lung cancer development.

To further investigate the influence of smoking reduction, we modelled the amount of change in smoking as a continuous variable using restricted cubic splines. If the patients had − 100%, it meant that they quit smoking. Four knots were selected based on a model comparison using the Akaike Information Criterion. We performed cubic splines for the change in smoking with knots at the 5th, 35th, 65th, and 95th percentiles of our sample distributions based on Harrell’s suggested knot locations. We then calculated the linearity of the association between the amount of change in smoking and the incidence of lung cancer by testing whether the coefficients associated with the nonlinear components were equal to zero.

Additionally, we used the Fine and Gray method to calculate the sub-distribution hazard ratios (subHRs) for the incidence of lung cancer to account for competing risks due to mortality [28].

All statistical analyses were performed using SAS version 9.4 (SAS Institute Inc., Cary, NC, USA) and R version 4.0.3 (R Foundation for Statistical Computing, Vienna, Austria).

## Results

Of the 16,832 patients with COPD (median age, 64 years; 87.3% men), 7,859 (46.7%) continued smoking, 2,823 (16.8%) reduced smoking, and 6,150 (36.5%) quit smoking after their respective COPD diagnoses. Among the reducers, 1,498 and 1,325 reduced daily smoking amounts by 20–50% and > 50%, respectively. Compared with sustainers, reducers and quitters were more likely to be older, have more comorbidities, and have a severe exacerbation history, but they were less frequent drinkers, had higher physical activity levels, and had a lower smoking pack-years at Exam 1 (Table 1).

**Table 1:** Characteristics of study participants (*N* = 16,832)

	Sustainer	Reducer	Quitter	*P*-value
	*N* = 7,859	*N* = 2,823	*N* = 6,150	
**Age, years, mean (SD)**	62.0 (10.6)	63.4 (11.0)	65.6 (10.5)	< 0.001
**Sex (%)**				0.068
Male	6,876 (87.5)	2,428 (86.0)	5,393 (87.7)	
Female	983 (12.5)	395 (14.0)	757 (12.3)	
**Area, metropolitan (%)**	4,515 (57.5)	1,606 (56.9)	3,454 (56.2)	0.312
**Income (%)**				< 0.001
Medical Aid	571 (7.3)	211 (7.5)	301 (4.9)	
≤ 30th	1,863 (23.7)	685 (24.3)	1,374 (22.3)	
31st – 70th	2,763 (35.2)	977 (34.6)	2,138 (34.8)	
> 70th	2,558 (32.5)	910 (32.2)	2,230 (36.3)	
Unknown	104 (1.3)	40 (1.4)	107 (1.7)	
**BMI (%)**				< 0.001
Underweight (< 18.5 kg/m^2^)	602 (7.7)	232 (8.2)	410 (6.7)	
Normal (18.5–23 kg/m^2^)	3,142 (40.0)	1,184 (41.9)	2,290 (37.2)	
Overweight (23–25 kg/m^2^)	1,778 (22.6)	591 (20.9)	1,444 (23.5)	
Obesity (> 25 kg/m^2^)	2,337 (29.7)	816 (28.9)	2,005 (32.6)	
Unknown	0 (0.0)	0 (0.0)	1 (0.0)	
**Drinking status (%)**				< 0.001
No	3,268 (41.6)	1,284 (45.5)	3,314 (53.9)	
Yes	4,588 (58.4)	1,538 (54.5)	2,834 (46.1)	
Unknown	3 (0.0)	1 (0.0)	2 (0.0)	
**Regular physical activity (%)**	1,000 (12.7)	405 (14.3)	907 (14.7)	0.002
**Pack-year at Exam 1, mean (SD)**	15.0 (7.3)	16.9 (6.8)	14.3 (7.6)	< 0.001
**Daily smoking intensity at Exam 1 (%)**				< 0.001
Light	1,592 (20.3)	419 (14.8)	1,767 (28.7)	
Moderate	4,515 (57.5)	1,541 (54.6)	3,421 (55.6)	
Heavy	1,752 (22.3)	863 (30.6)	962 (15.6)	
Medication*				
ICS (%)	711 (9.0)	299 (10.6)	689 (11.2)	< 0.001
LABA (%)	1,004 (12.8)	395 (14.0)	865 (14.1)	0.055
LAMA (%)	1,305 (16.6)	524 (18.6)	1,344 (21.9)	< 0.001
Severe exacerbation (%)*	479 (6.1)	215 (7.6)	665 (10.8)	< 0.001
Comorbidities*				
CCI, mean (SD)	2.4 (2.2)	2.5 (2.3)	2.7 (2.4)	< 0.001
Pulmonary tuberculosis (%)	175 (2.2)	82 (2.9)	185 (3.0)	0.01
Interstitial lung disease (%)	112 (1.4)	42 (1.5)	155 (2.5)	< 0.001
Bronchiectasis (%)	208 (2.6)	78 (2.8)	272 (4.4)	< 0.001
Pneumonia (%)	796 (10.1)	314 (11.1)	898 (14.6)	< 0.001

BMI, body mass index; COPD, chronic obstructive pulmonary disease; CCI, Charlson comorbidities index; ICS, inhaled corticosteroid; LABA, long-acting beta-2 agonist; LAMA, long-acting muscarinic antagonist

All variables were assessed at Exam 2, except for smoking pack-year and daily smoking intensity, which were assessed at Exam 1

* These variables were assessed within 1 year of Exam 2

**Fig. 2:**
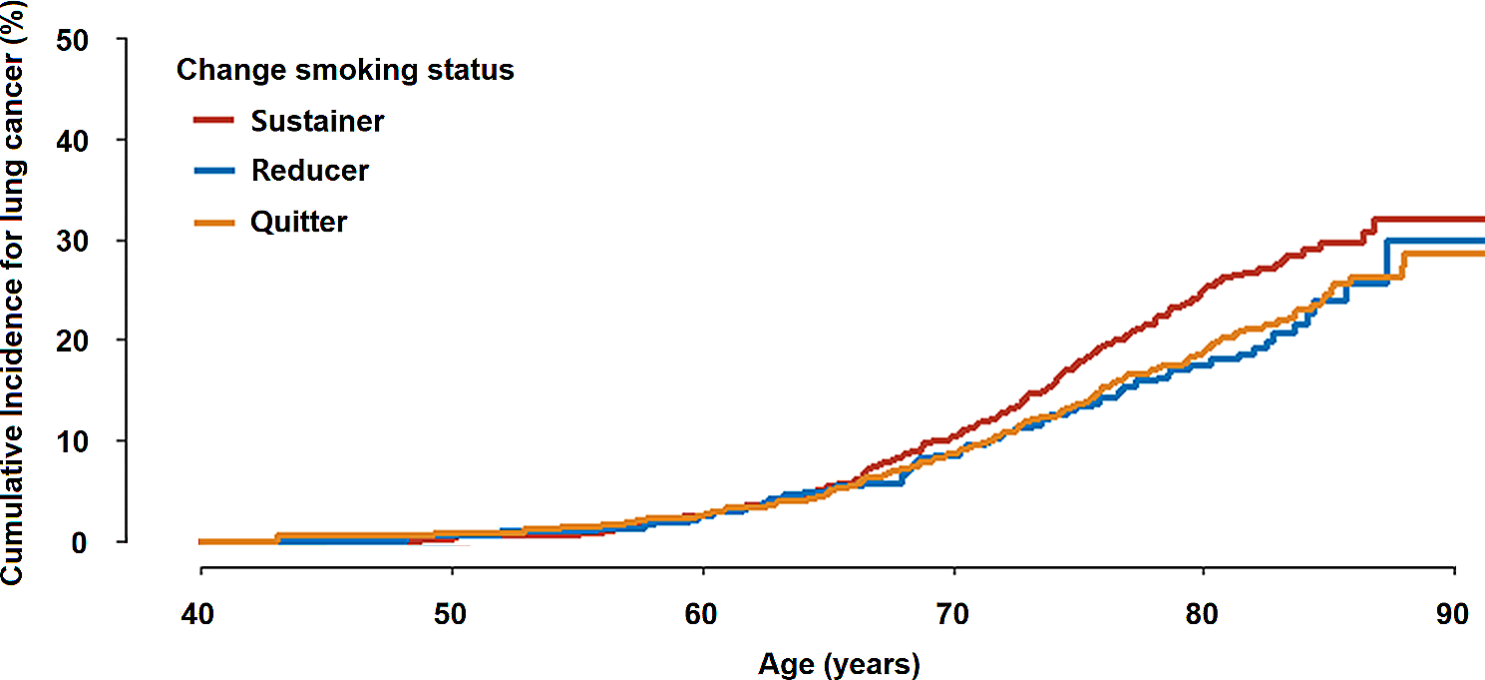
Kaplan Meier curve for incidence of lung cancer. Age as time scale

During a median follow-up of 3.94 years, a total of 469 new lung cancer diagnoses were made. The cumulative incidence of lung cancer was the highest among sustainers, followed by reducers and quitters (Fig. 2). The multivariable-adjusted HRs (95% CIs) for lung cancer were 0.74 (0.56, 0.98), and 0.78 (0.64, 0.96) in reducers and quitters, respectively. The results were similar when competing risk analysis was performed (Table 2).

**Table 2:** Hazard ratio (HR) with 95% confidence interval (CI) for incident lung cancer associated with change of smoking status

	No of cases	100-person year	Adjusted HR (95% CI)	Adjusted subHR* (95% CI)
Sustainer	225	0.75	*Reference*	*Reference*
Reducer	68	0.61	**0.74 (0.56, 0.98)**	**0.74 (0.57, 0.97)**
20–50% reducer	39	0.67	0.84 (0.60, 1.18)	0.83 (0.59, 1.17)
Over 50% reducer	29	0.55	**0.64 (0.64, 0.95)**	**0.64 (0.43, 0.95)**
Quitter	176	0.73	**0.78 (0.64, 0.96)**	**0.78 (0.64, 0.95)**

Adjusted for age, sex, BMI, residential area, income, regular physical activity, pack-years (Exam 1), ICS within 1 year of Exam 2, severe exacerbation within 1 year of Exam 2, and comorbidities within 1 year of Exam 2.

* Sub-distribution hazard ratios (subHRs) for lung cancer were modelled with mortality as a competing risk.

BMI, body mass index; COPD, chronic obstructive pulmonary disease; ICS, inhaled corticosteroids

**Fig. 3:**
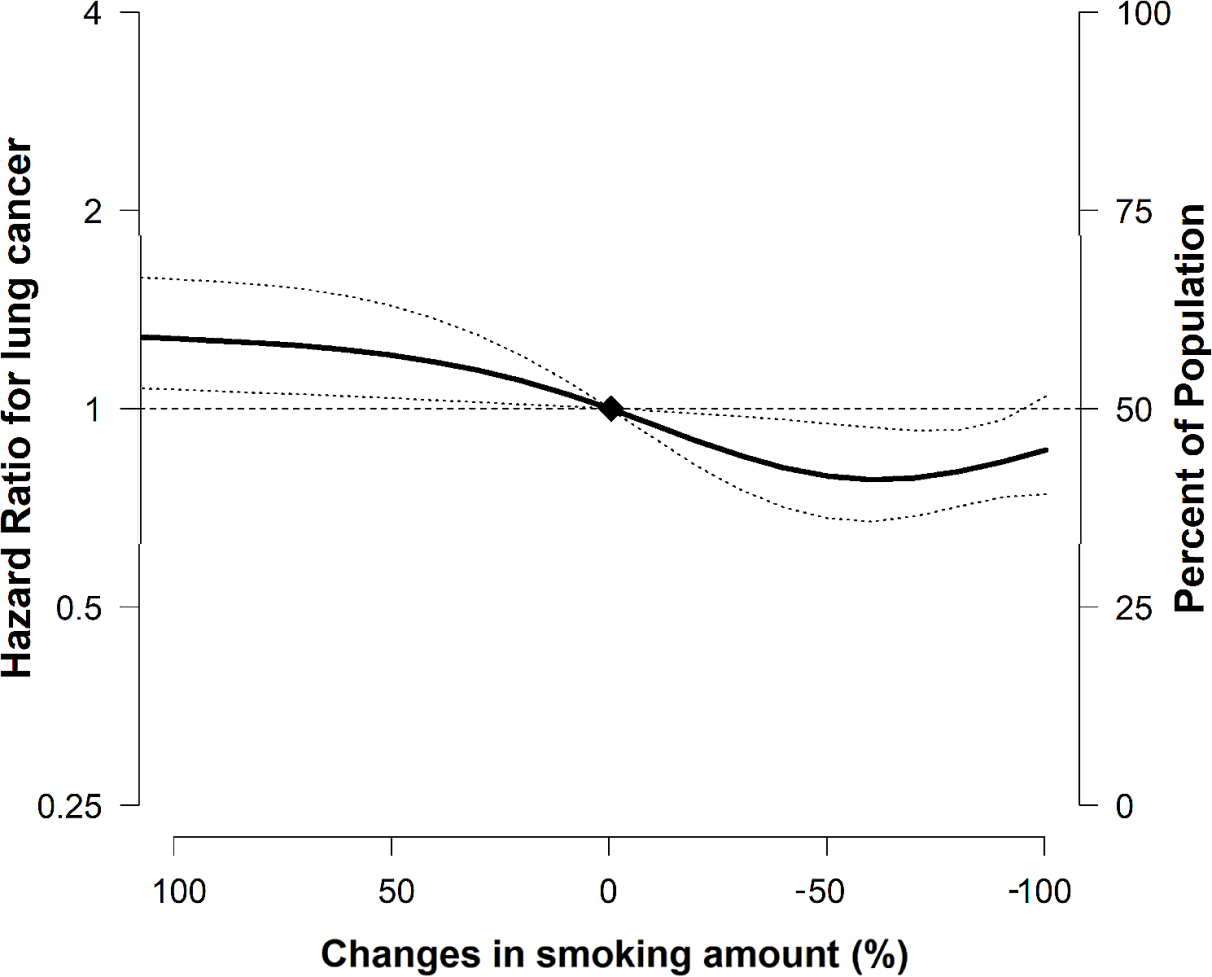
Multivariable-adjusted hazard ratios (95% CI) for incidence of lung cancer according to amount of smoking change. The curves represent the adjusted odds ratios (solid lines) and their 95% confidence intervals (dashed lines) for the incidence of lung cancer based on restricted cubic splines for the amount of smoking change with knots at the 5th, 35th, 65th, and 95th percentiles of their sample distributions. The reference value (diamond dots) was set to zero, which did not change. Quitter group was categorized in -100%

When the effect of smoking reduction was analyzed in subcategories by the amount of reduction, the multivariable-adjusted HRs (95% CIs) for lung cancer were 0.84 (0.60, 1.18), and 0.64 (0.64, 0.95) in 20–50% reducers and >50% reducers, respectively, compared with those who continued smoking (Table 2). These findings remained consistent after conducting a competing risk analysis. In the restricted cubic spline model, the incidence of lung cancer showed a decreasing trend with a decreasing amount of smoking (P for linearity < 0.01, Fig. 3).

An association between smoking reduction or cessation and the risk of lung cancer was observed in all analysed subgroups. In particular, the protective effect was stronger in males than in females (P for interaction = 0.02) (Table 3).

**Table 3:** Subgroup analysis

	No of cases (100-person year)	Adjusted HR* (95% CI)	P for interaction
**Age**			0.77
**≤ 65**			
Continue	54 (0.3)	Reference	
Over 20% Reducer	17 (0.3)	0.79 (0.45, 1.38)	
Quit	26 (0.3)	0.77 (0.48, 1.23)	
**> 65**			
Continue	171 (1.4)	Reference	
Over 20% Reducer	51 (1.0)	**0.72 (0.53, 0.99)**	
Quit	150 (1.1)	**0.77 (0.62, 0.97)**	
**Sex**			0.02
**Male**			
Continue	210 (0.8)	Reference	
Over 20% reducer	56 (0.6)	**0.67 (0.50, 0.91)**	
Quit	167 (0.8)	**0.79 (0.64, 0.98)**	
**Female**			
Continue	15 (0.4)	Reference	
Over 20% reducer	12 (0.8)	1.36 (0.61, 3.01)	
Quit	9 (0.3)	0.59 (0.25, 1.42)	
**Income**			0.89
**Medical aid**			
Continue	7 (0.3)	Reference	
Over 20% reducer	3 (0.4)	1.02 (0.21, 5.08)	
Quit	2 (0.2)	**0.07 (0.01, 0.71)**	
**≤ 30th**			
Continue	48 (0.7)	Reference	
Over 20% reducer	13 (0.5)	0.65 (0.35, 1.22)	
Quit	36 (0.7)	0.87 (0.56, 1.36)	
**31st – 70th**			
Continue	85 (0.8)	Reference	
Over 20% reducer	22 (0.6)	0.63 (0.39, 1.01)	
Quit	61 (0.7)	0.72 (0.51, 1.01)	
**> 70th**			
Continue	81 (0.8)	Reference	
Over 20% reducer	30 (0.9)	0.92 (0.60, 1.41)	
Quit	75 (0.9)	0.85 (0.61, 1.17)	
**BMI**			0.89
**Underweight**			
Continue	20 (0.9)	Reference	
Over 20% reducer	8 (0.9)	0.88 (0.38, 2.03)	
Quit	13 (0.8)	0.69 (0.33, 1.43)	
**Normal**			
Continue	107 (0.9)	Reference	
Over 20% reducer	32 (0.7)	0.72 (0.48, 1.07)	
Quit	74 (0.8)	**0.74 (0.54, 0.99)**	
**Overweight**			
Continue	54 (0.8)	Reference	
Over 20% reducer	12 (0.5)	0.61 (0.33, 1.16)	
Quit	51 (0.9)	0.97 (0.66, 1.44)	
**Obesity**			
Continue	44 (0.5)	Reference	
Over 20% reducer	16 (0.5)	0.93 (0.52, 1.66)	
Quit	38 (0.5)	0.75 (0.48, 1.17)	
**Smoking status at Exam 1**			0.45
**Light-Moderate**			
Continue	193 (0.8)	Reference	
Over 20% reducer	58 (0.8)	0.79 (0.58, 1.06)	
Quit	157 (0.8)	**0.78 (0.63, 0.97)**	
**Heavy**			
Continue	32 (0.5)	Reference	
Over 20% reducer	10 (0.3)	0.52 (0.25, 1.07)	
Quit	19 (0.5)	0.82 (0.45, 1.49)	
**CCI¶**			0.88
**≤ 1**			
Continue	91 (0.7)	Reference	
Over 20% reducer	24 (0.5)	0.67 (0.42, 1.06)	
Quit	57 (0.6)	0.73 (0.52, 1.01)	
**> 1**			
Continue	134 (0.8)	Reference	
Over 20% reducer	44 (0.7)	0.78 (0.55, 1.10)	
Quit	119 (0.8)	0.82 (0.63, 1.05)	
**Severe exacerbation¶**			0.43
**No**			
Continue	216 (0.8)	Reference	
Over 20% reducer	64 (0.6)	**0.73 (0.55, 0.97)**	
Quit	154 (0.7)	**0.76 (0.61, 0.93)**	
**Yes**			
Continue	9 (0.5)	Reference	
Over 20% reducer	4 (0.5)	0.89 (0.27, 2.99)	
Quit	22 (0.9)	1.39 (0.61, 3.16)	

All P for interactions were not statistically significant (*P* > 0.05), except for sex

* Adjusted for age, sex, BMI, residential area, income, regular physical activity, pack-years (Exam 1), ICS within 1 year of Exam 2, severe exacerbation within 1 year of Exam 2, and comorbidities within 1 year of Exam 2

¶ Variables were assessed within 1 year of Exam 2

BMI, body mass index; ICS, inhaled corticosteroid; COPD, chronic obstructive pulmonary disease; HR, hazard ratio; CI, confidence interval; CCI, Charlson Comorbidity Index

## Discussion

In this nationwide population-based cohort with comprehensive data on health status and medical service utilization of the entire Korean population, smoking reduction and smoking cessation were independently associated with a lower risk of lung cancer development in patients with COPD who smoked less than 30 pack-years, after adjusting for major confounders, including cumulative smoking amount and sociodemographic factors. This association was consistent across all the subgroups, with a less pronounced effect observed in females than in males. In particular, we found a decreasing trend of lung cancer risk with a decreasing amount of smoking. Our results underscore that smoking cessation should remain the most effective way to reduce the risk of lung cancer development, but smoking reduction may be used as an adjusted strategy to reduce the risk of lung cancer in patients with COPD, particularly in those who have smoked less than 30 pack-years and are unable to quit smoking immediately.

Importantly, our study found that the risk of lung cancer development in patients with COPD decreased when they merely reduced their smoking amount, which is in line with a previous reports based on population-based samples [5, 6, 29, 30]. A previous large population-based Danish study (*n* = 19,714) demonstrated a decrease in the lung cancer risk with smoking reduction by 27% compared to the persistent heavy smokers for up to 31 years of follow-up [8]. In our study, the benefit of smoking reduction and cessation in lowering lung cancer risk was clear with a relatively short follow-up period (median, 4 years). This result encourages patients with COPD who are current smokers but are unable to stop smoking immediately in real-world practice to gradually reduce their smoking amount. Gradual smoking reduction could serve as an intermediate step towards achieving complete smoking cessation. Although abrupt smoking cessation is more likely to result in lasting abstinence than a gradual decrease [31], various real-world barriers, such as social and environmental factors, could make it impractical or challenging for certain groups of smokers to quit smoking abruptly [32]. Moreover, smokers who reduce their smoking before quitting are more likely to quit smoking successfully than those who do not [33]. In particular, a greater reduction in cigarettes smoked per day increased the likelihood of future cessation [34]. Therefore, for patients with COPD who are unable to quit smoking immediately, a gradual reduction in the amount of smoking might have a positive impact on lowering the risk of developing lung cancer. However, it should be acknowledged that smoking cessation is the cornerstone of preventing smoking-related cancer development as well as mitigating the progression of COPD, which will inevitably progress with accelerated lung function decline and increased exacerbation with continuous smoking [10, 35].

Several factors are thought to play a role in this observation that smoking reduction and cessation confers a decreased risk of lung cancer in patients with COPD. Possible biological mechanisms have also been suggested. Smoking reduction and cessation could lower exposure to carcinogenic substances, reduce oxidative stress, and have the potential to mitigate or reverse epigenetic alterations resulting from tobacco use [36–38]. Changes in smoking habits may also contribute to the restoration of immune function [39]. Genes responsible for the antitumor response are hypermethylated in patients with COPD who smoke, suggesting reduced infiltration of immune cells against tumor [40]. Moreover, patients with COPD who changed their behavior to smoking reduction or cessation were more likely to be concerned about their health status. In our exploratory analysis, quitters exhibited the lowest alcohol consumption and highest engagement in regular physical activity. In addition, a higher rate of severe exacerbation in the previous year and a higher disease burden in quitters could have motivated them to quit smoking. Although further research is needed to establish the causality and significance of these factors in the context of smoking reduction and lung cancer risk in patients with COPD, it is important to provide information that motivates behavioral changes to quit or reduce smoking.

The subgroup analysis revealed that the benefits of smoking reduction and cessation on lung cancer risk were less evident in females than in males. Several studies have shown an increased susceptibility to cigarette smoke in female smokers compared with male smokers, including a higher risk of airflow obstruction development and hospitalization for COPD with accelerated lung function decline in females [41–43]. However, there are conflicting data regarding sex differences in smoking and the incidence of lung cancer. Several case-control and cohort studies have shown that female smokers have a higher risk of lung cancer than male smokers with the same smoking quantity [44–47], whereas other cohort studies have shown a similar incidence of lung cancer with comparable smoking histories [48, 49]. Few studies have investigated whether the impact of smoking reduction and cessation on lung cancer risk differs between females and males, especially in smokers using less than 30 pack-years [50]. Given the variations in smoking patterns as well as biological differences between males and females could contribute to the differences in lung cancer risk reduction, this discrepancy observed in the subgroup analysis requires validation in further studies.

To the best of our knowledge, this study is the first to show the immediate impact of smoking reduction and cessation on lowering lung cancer risk in patients with COPD with a smoking history of less than 30 pack-years. We also demonstrated a decreasing trend of lung cancer risk with a decreasing amount of smoking among reducer and sustainer group, although risk in reducers were not statistically different compared to that in complete quitters. Estimates derived from national insurance data may guarantee the representativeness of the entire COPD population in South Korea. However, this study also has several limitations. First, data of spirometric measurements are not available in K-NHIS data. Thus, the COPD diagnosis was based on administrative data rather than clinical diagnosis using spirometry, which may have resulted in misclassification bias. Nevertheless, several previous studies based on claims data have adopted this operational definition of COPD [3, 4, 21, 22]. Second, smoking habits were self-reported through questionnaires rather than confirmed through biochemical methods, such as urine cotinine levels, which may lead to recall, misclassification, and measurement errors. Third, we lacked information on the histologic types of lung cancer. Additional efforts are necessary to fully elucidate the effect of smoking reduction and cessation on the development of lung cancer in patients with COPD across several lung cancer subtypes [9]. Fourth, the median 4-year follow-up period in this study was relatively short, and the long-term protective effect of smoking reduction (i.e., continued smoking, albeit in a reduced amount) cannot be guaranteed. Further longitudinal studies are necessary to validate our findings. Therefore, healthcare providers must encourage patients with COPD to stop smoking in every clinic visit. Fifth, our study population may not represent the entire COPD population, as we focused COPD patients who utilized health care services including the prescription of COPD medication and who participated the national health screening examinations. The participation rate for the health screening exam is 74% despite its being free-of-charge. Lastly, the majority of study participants (87.3%) were males, which raises concerns regarding the generalizability of our findings to female patients.

## Conclusions

Our study highlights the importance of smoking reduction and smoking cessation in lowering the risk of lung cancer development in patients with COPD who smoked less than 30 pack-years. Reducing the smoking amount might be a starting point for individuals who struggle to quit abruptly with active encouragement of smoking cessation in every clinic, as smoking cessation is the single most effective way not only to reduce lung cancer development and mortality in patients with COPD, but also to ameliorate the natural course of COPD.

## Data Availability

The data are available from the Korean National Health Insurance Sharing Service (NHISS; https://nhiss.nhis.or.kr/) database, which is open to researchers on request with approval by the Institutional Review Board.
